# The Association between High Body Mass Index and Early Clinical Outcomes in Patients with Proximal Femur Fractures

**DOI:** 10.3390/jcm9072076

**Published:** 2020-07-02

**Authors:** Michael Müller, Alexander Gutwerk, Frederik Greve, Lisa Völker, Michael Zyskowski, Chlodwig Kirchhoff, Peter Biberthaler, Dominik Pförringer, Karl Braun

**Affiliations:** 1 Klinik und Poliklinik für Unfallchirurgie, Klinikum rechts der Isar, Technische Universität München, 81675 München, Germany; frederik.greve@mri.tum.de (F.G.); lisa.voelker@mri.tum.de (L.V.); michael.zyskowski@mri.tum.de (M.Z.); chlodwig.kirchhoff@mri.tum.de (C.K.); peter.biberthaler@mri.tum.de (P.B.); dominik.pfoerringer@mri.tum.de (D.P.); karl.braun@charite.de (K.B.); 2 Orthopädie, Sport- & Unfallklinik, Ev.-Luth. Diakonissenanstalt, 24939 Flensburg, Germany; gutwerkal@diako.de; 3 Centrum für Muskuloskeletale Chirurgie, Charité Universitätsmedizin Berlin, 13353 Berlin, Germany

**Keywords:** obesity, body mass index, BMI, proximal femoral fracture, hip fracture, arthroplasty, complications, functional outcomes

## Abstract

Background: Fractures of the proximal femur constitute daily work in orthopedic trauma surgery. With the continuous increase of obesity in the general population, surgeons face several known technical challenges. The aim of this study was to investigate the association of high body mass index (BMI) in patients with proximal femur fractures with intra- and postoperative adverse events, as well as with functional outcomes after successful surgery. Methods: In this retrospective, single-center cohort study, 950 patients who sustained a fracture of the proximal femur (femoral neck fracture or trochanteric fracture) and underwent surgical treatment at our level I trauma center between 2003 and 2015 were included. Patient-specific data were obtained in regard to demographics, comorbidities, and fracture morphology. In-hospital postoperative complications (i.e., need for revision surgery, wound site infection, pneumonia, urinary tract infection, necessary transfusion, and deep-vein thrombosis) were analyzed, along with the length of hospitalization and overall mortality rate. Functional outcome was assessed using the Barthel index and the patient’s ability to walk on crutches. Mortality rate and need for revision surgery were assessed over a two-year time period. Any adverse event was correlated to one of the four WHO’s BMI groups. Results: The cohort included 80 (8.4%) underweight patients, 570 (60.0%) normal weight patients, 241 (25.4%) overweight patients, and 59 (6.2%) obese patients. We found more femoral neck fractures (506, or 53%) than trochanteric fractures (444, or 47%). In bivariate analysis, no significant difference was found in regard to overall mortality or postoperative complications. Hospitalization time (LOS) differed between the underweight (12.3 ± 4.8 days), normal (13.6 ± 7.8 days), overweight (14.2 ± 11.7 days), and obese patients (16.0 ± 9.7 days) (*p* = 0.040). Operation time increased stepwise with increasing BMI: underweight = 85.3 ± 42.9 min; normal weight = 90.2 ± 38.2 min; overweight = 99.9 ± 39.9 min; obese = 117.2 ± 61.5 min (*p* < 0.001). No significant difference was found by analyzing functional outcomes. However, patients with intermediate BMI levels (18.5–30 kg/m^2^) tended to achieve the best results, as represented by a higher Barthel index score and the patient’s ability to walk on crutches. Conclusion: Increased BMI in patients with proximal femur fractures is associated with both longer operation time and length of hospitalization (LOS). Postoperative mobilization and functional outcomes appear to follow a reversed J-curve distribution (with overweight patients showing the best functional results), whereas both obese and underweight patients have associated poorer function.

## 1. Introduction

Proximal femur fractures are among the most common fracture entities in elderly patients and determine the everyday work of an orthopedic surgeon. They represent the most common non-vertebral fractures in elderly men and the second most common non-vertebral fractures in elderly women, following wrist fractures [1]. In Germany, the incidence of proximal femur fractures is estimated to be above 100,000 patients per year [2]. The ratio between femoral neck fractures and trochanteric fractures is 54% vs. 46% [3]. Epidemiological observations predict a further increase of the incidence of proximal femur fractures with an aging society worldwide, thus stressing their significance [4]. Another development is the worldwide progression of obesity becoming a global epidemic, especially in industrialized countries [5]. 

Several studies have shown that low body weight is associated with low bone density and a higher risk of falling [6]. A body mass index (BMI) below 20 kg/m^2^ is associated with a significantly higher relative risk for proximal femur fractures [7,8]. Overweight patients with proximal femur fractures are thought to have poorer postoperative outcomes; however, scientific data remain inconclusive to this day. While the one-year survival rate after hip fracture seems to be higher for patients with a BMI of 26 and above [9], the negative effects of increased body weight are described in regard to postoperative complications and the length of stay in the early postoperative period [10]. Other studies contradict these results and show no difference in hospitalization or postoperative complications in patients with a high BMI [11,12]. Furthermore, some studies even describe a protective effect of an overweight body constitution (BMI 25–29.9 kg/m^2^) when compared to underweight patients with regard to postoperative complications after elective or trauma-related hip surgery [13].

Over the last decades, a constant increase of the prevalence of overweight and obese patients has been found, especially in Western societies [14]. Therefore, it can be assumed that an increasing number of overweight patients with proximal femur fractures will challenge the surgeon in the future. In order to optimize the surgical treatment of obese patients, it is crucial to know the specific risks and complications that can appear in this collective. A review of the literature shows that many studies focus on outcomes after elective hip arthroplasty rather than fractures of the proximal femur. Thus, the aim of the present study was to evaluate the association between BMI and postoperative complications, functional outcomes, length of hospitalization, and mortality after proximal femur fractures with a focus on the early postoperative period.

## 2. Methods

We performed a single-center, retrospective study analyzing patients who underwent surgery after proximal femur fractures in our Orthopedic Trauma Surgery Department at the “Klinikum rechts der Isar”, University Hospital of the Technical University of Munich (TUM), in the period from December 2003 to June 2015. Approval for the study was obtained from the Ethics Committee of the Medical Faculty, Klinikum rechts der Isar, Technical University of Munich, Germany (study number 409/15s).

### 2.1. Study Population

Patients were identified using a computer-based data management program (SAP), searching for all operatively treated patients diagnosed with ICD-10 codes S72.0–S72.2. All femoral neck fractures and trochanteric fractures were included, whereas conservatively treated fractures and fractures of the femoral head were excluded. Further exclusion criteria were revision surgery, severely injured patients, or patients with concomitant fractures requiring multiple surgical treatments. Furthermore, patients with tumor-related pathologic fractures were not analyzed. To determine nutritional status, we calculated the BMI. We classified the patients according to the criteria of the World Health Organization, which has four different categories: underweight (UW) (BMI < 18.5 kg/m^2^), normal weight (NW) (BMI 18.5–24.9 kg/m^2^), overweight (OW) (BMI 25.0–29.9 kg/m^2^), and obese (OB) (BMI ≥ 30.0 kg/m^2^) [15].

### 2.2. Data Collection

Data were recorded from the time of the patient’s admission until their discharge. The documentation provided by the surgeons, anesthesiologists, nurses, and physiotherapists involved was used. No follow-up examinations were performed. The body weight, height, and the American Society of Anesthesiologists (ASA) scores for the patients were assessed from the documentation provided by the anesthesiologists. Furthermore, preoperatively collected data contained demographics, type of fracture, and standardized assessment of pre-existing medical conditions (e.g., diabetes, arterial hypertension, coronary heart disease, hypothyroidism, dementia, stroke, chronic kidney disease, tumor disease, or substance addiction). Substance addiction was defined as abuse of nicotine, alcohol, or drugs. Complications, such as the need for revision surgery or dislocation of the arthroplasty, were obtained from our computer-based documentation system. Complications treated in other hospitals could not be respected in our analysis.

### 2.3. Surgical Therapy

The type of surgical treatment depended on the type of fracture. Trochanteric fractures were treated with a cephalomedullary nailing system (Proximal Femoral Nail Antirotation system, Synthes GmbH, Oberdorf, Switzerland; or TRIGEN INTERTAN Intertrochanteric Antegrade Nail system, Smith and Nephew Inc., Cordova, USA) in the majority of cases. A minority of trochanteric fractures were treated with a dynamic hip screw (DHS Dynamic Hip System, DePuy Synthes GmbH, Oberdorf, Switzerland). Both interventions were performed using a fracture table with axial traction. For cephalomedullary nailing, a lateral approach was used, with an entry point at the tip of the greater trochanter. Femoral neck fractures were treated with a dynamic hip screw or arthroplasty (total hip arthroplasty (THA) or hemiarthroplasty (HA)), depending on the patient’s individual needs (Zimmer Biomet, Warsaw, USA). THA and HA were performed using the anterolateral Watson–Jones approach. Details about the operation time were taken from the nurses’ documentation. The operation time was defined as the time from incision to suture. Patients treated with cephalomedullary nails or hip arthroplasty were immediately allowed full weight bearing movement. Patients treated with DHS followed a 15 kg weight bearing limitation. Range of motion was limited to 90° flexion postoperatively for all patients.

### 2.4. Outcome Parameters

We evaluated the following patient-based risk factors at the time of admission: age, gender, ASA score [16], total number of comorbidities, and total number of medications taken. The variable number of comorbidity did not contain any preexisting medical conditions, rather only the 10 predefined diseases that were most commonly found in the anamnesis. These predefined comorbidities were: diabetes mellitus, arterial hypertension, coronary heart disease, addictive disorders, hypothyroidism, osteoporosis, dementia, past cerebral ischemia, and kidney failure. Complications were recorded, such as the need for revision surgery, dislocation of arthroplasty, postoperative blood loss and subsequent need for transfusion, wound site or implant infection, pneumonia, deep vein thrombosis, or pulmonary embolism. The principal outcome measures were the length of stay (LOS), mortality rate, operation time, and postoperative Barthel index score prior to discharge. The Barthel index is an ordinal score ranging from 0 to 100, representing the performance in activities of daily living. It is frequently used to assess a patient’s capabilities in order to resume their activities of daily life [17]. The level of mobilization was classified using the documentation of physiotherapists, with the ability to walk on crutches independently being the most important outcome parameter. Mortality and necessary revision surgery were documented during the first two postoperative years. Furthermore, we investigated how patients with femoral neck fractures and patients with trochanteric fractures differed in regard to their baseline characteristics and outcomes (LOS, mortality, mobilization). Risk for developing a femoral neck fracture rather than a trochanteric fracture was analyzed and adjusted for covariates such as BMI, age, gender, and osteoporosis. In this model, BMI was used as a continuous variable.

### 2.5. Statistics

For bivariate analyses, continuous variables were described with means ± standard deviations. Binary variables were compared with percentages in cross-tables. Differences between the four BMI groups were analyzed using the Kruskal–Wallis test for continuous variables (age, operation time, etc.) and Chi-square test for dichotomous variables. Additionally, linear or logistic multivariate regression analysis was performed in order to control confounding variables such as age, gender, fracture type, or ASA score. Continuous variables such as the operation time and LOS that did not show normal distribution were logarithmized for the linear regression. The level of significance was defined as *p* < 0.05. Statistics were calculated using SPSS (Version 22, IBM SPSS Statistics for Windows, Armonk, NY, USA).

## 3. Results

A total of 1368 patients with the defined ICD-10 code were identified. After correction for the exclusion criteria, 950 patients met our criteria for undergoing surgery related to proximal femur fractures between December 2003 and June 2015. In total, 637 (67.1%) patients were female and 313 (32.9%) patients were male. The mean age was 74.8 ± 13.51 years (range 23–99 years). Female patients were significantly older (77.4 ± 11.8 years) than male patients (69.5 ± 15.1 years) (*p* < 0.001). The mean BMI was 23.53 ± 4.17 kg/m^2^. Patients with a BMI < 18.5 showed the highest average age, however with no statistical significance (*p* = 0.089).

### 3.1. Type of Fracture

In total, 506 (53.3%) femoral neck fractures and 444 (46.7%) trochanteric fractures were observed. Femoral neck fractures were treated with either THA (*n* = 147; 29.1%), HA (*n* = 278; 54.9%), or dynamic hip screw (*n* = 81; 16.0%). Trochanteric fractures were treated with either a cephalomedullary nail (*n* = 398; 89.6%) or a dynamic hip screw (*n* = 46; 10.4%). Patients with trochanteric fractures were significantly older (76.1 ± 13.3 years) than those with femoral neck fractures (73.7 ± 13.6 years) (*p* = 0.02) (Table 1). There was no difference in the prevalence of osteoporosis. LOS did not differ between the groups (13.76 days vs. 13.78 days). Femoral neck fractures showed significantly higher in-hospital mortality (4.3%; *n* = 22) than trochanteric fractures (2.0%; *n* = 9) (*p* = 0.041). This bivariate analysis was confirmed by a logistic regression model controlled for covariates age, gender, and ASA score. Mortality OR for femoral neck fractures vs. trochanteric fractures was 2.483 (0.121-0.5.496 95% CI; *p* = 0.025).

The ratio of trochanteric fractures to femoral neck fractures increases with age, as seen in patients older than 85 years (*n* = 220), with a changed ratio of trochanteric fractures to femoral neck fractures of 53.6% (*n* = 118) to 46.4% (*n* = 102) being observed. A logistic regression analysis was performed to identify independent risk factors for having a trochanteric fracture rather than a femoral neck fracture (Table 2). Body mass index (OR 1.033, 95% CI, 1.002–1.066; *p* = 0.040) and age (OR 1.016, 95% CI, 1.006–1.027; *p* = 0.002) were significant risk factors when controlled for the covariates female gender and osteoporosis.

### 3.2. Differences between the BMI Groups

The mean BMI was 23.5 ± 4.2 kg/m^2^, with a minimum of 12.8 kg/m^2^ and a maximum of 48.4 kg/m^2^. The distribution among the BMI groups was: UW 8.4% (*n* = 80), NW 60% (*n* = 570), OW 25.4% (*n* = 241), OB 6.2% (*n* = 59). Table 3 shows the baseline characteristics and individual morbidities of the investigated patient collective for the four BMI groups. Significant differences between the groups were found regarding gender; preoperative ASA score; fracture type; and prevalence of diabetes, hypertension, and substance addiction, as well as the number of medications taken.

**Figure 1:**
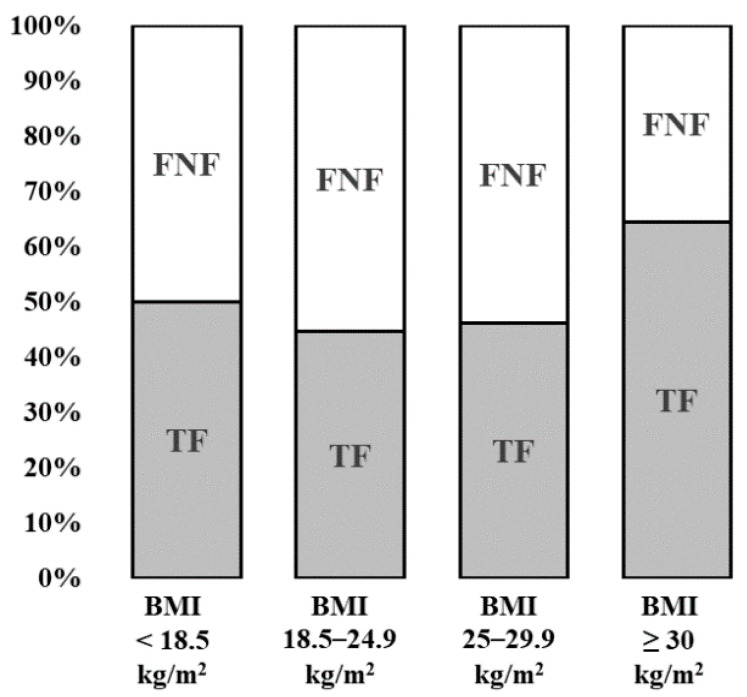
Ratio of femoral neck fractures (FNFs) to trochanteric fractures (TFs), depending on the body mass index (BMI) classification.

UW patients were more likely to be female compared to NW, OW, or OB patients. There was a significant difference between the different groups regarding their pre-existing comorbidities. The total number of comorbidities was significantly higher in OB patients (3.10 ± 1.6) than in UW and NW patients (2.47 ± 1.9 and 2.38 ± 1.8, respectively). This was also reflected in the preoperative ASA scores. OB patients had a significantly higher ASA score than all other BMI groups. The prevalence of both diabetes mellitus and arterial hypertension showed a significant increase with increasing BMI. This trend could also be seen in the prevalence of coronary heart disease without reaching the level of significance (*p* = 0.053). UW patients had a 50/50 ratio of femoral neck fractures to trochanteric fractures. With increasing BMI, this ratio shifted in favor of trochanteric fractures, reaching a maximum of 64.4% in the OB patients (Figure 1).

The overall in-hospital mortality was 3.3% (*n* = 31). Significant differences between the groups were not identified. 

No significant differences were found regarding postoperative complications between the different BMI groups (Table 4). However, a trend was noted in regard to postoperative infections of the wound site with higher BMI. Postoperative anemia with indication for blood transfusion appeared to be less frequent in both OW and OB patients as compared to the other two groups. There was a positive (but insignificant) association of higher BMI with the need for revision surgery. The overall revision rate within the first two years was 8.4% (*n* = 80). The reasons for revision were relevant postoperative hematoma, deep wound site infection, and infection or dislocation of the arthroplasty. The overall incidence of all dislocations in THAs or HAs was 2.4% (*n* = 10/415). OB patients had slightly more dislocations. The results had no significance (UW: *n* = 3, 3.8%; NW: *n* = 15, 2.6%; OW: *n* = 5, 2.1%; OB; *n* = 2, 3.4%; *p* = 0.846). Implant failure is rare and was only reported in 12 cases (1.3%).

Significant differences between the groups were found for operation time and the overall postoperative LOS. The operation time was significantly less in patients with reduced BMI. The operation time was 37% longer for OB patients compared to UW patients (117.2 ± 61.5 min vs. 85.3 ± 42.9 min) (*p* < 0.001). NW patients had intermediate operation times (90.2 ± 38.2 min).

The mean postoperative LOS in the hospital was 13.7 ± 8.9 days. OB patients had a longer postoperative LOS (16 ± 9.7 days), whereas the OW and NW patients had a shorter postoperative LOS (14.2 ± 11.7 days and 13.6 ± 7.8 days, respectively); UW patients had a postoperative LOS of only 12.3 ± 4.8 days. Results from bivariate analyses were verified in a multivariate analysis adjusted for age, gender, ASA score, and fracture type (Table 5).

**Figure 2:**
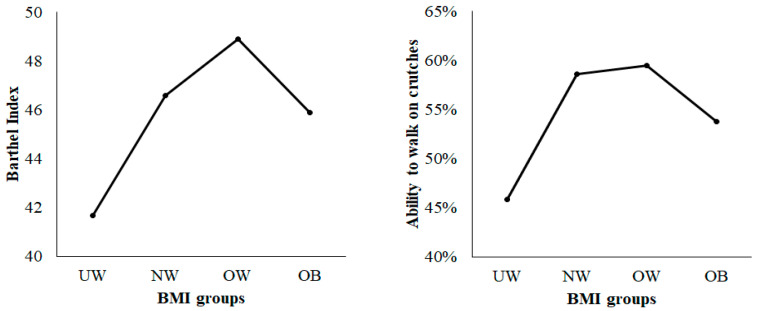
Line graph with reversed J-curve shape, demonstrating the functional status represented by the Barthel index score and mobilization on crutches for the BMI groups (UW = underweight; NW = normal weight; OW = overweight; OB = obese).

Regarding the mobility on forearm crutches, we found an advantage for the patients with intermediate BMI levels (18.5–29.9 kg/m^2^; NW and OW patients). Overall, 58.6% of these NW and 59.5% of OW patients were mobile on crutches. However, only 53.8% of OB patients and only 45.9% of UW patients reached mobility on crutches. The level of significance for these findings was missed (*p* = 0.098). Simultaneous distributions were seen regarding the maximum postoperative Barthel index score. The highest scores of 46.6. and 48.9 were within the NW and OW groups, while the UW and OB groups had lower scores of 41.7 and 45.0 (Figure 2).

## 4. Discussion

The aim of this study was to assess early postoperative outcomes in patients with proximal femur fractures and to determine if there was an association between body mass index and adverse events. One clear finding in our study was the longer operation time and longer LOS with increasing BMI. The duration of the surgery was longer in OW and OB patients compared to the other two groups. This confirms the data of other studies [18]. One reason might be the time-intensive surgical preparation through hypertrophic subcutaneous soft tissue during the approach, challenging the surgeon. Furthermore, a longer overall operation time was described due to prolonged anesthesia time [19]. According to Meller et al., this also increases the costs of care in OB patients [20].

Length of stay increased stepwise with higher BMI values. Mean LOS was 4 days longer for OB patients than for UW patients. This effect has been described by Hauck et al. [21] and Zizza et al. [22], however the reason remains unclear. The mean LOS (13.7 ± 8.9 days) was longer compared to other studies [12,13]. This might be due to the older study population and non-elective nature of the surgery performed. Anis et al. found that non-elective THAs had significantly longer operation times, longer LOS, and higher complication rates than elective THAs [23]. The reason for the prolonged hospitalization of OB patients was most probably delayed mobilization. Bryant et al. showed a delayed time until the first mobilization out of bed for OB patients [24]. Our findings are in line with these results.

There was no significant difference in in-hospital complications between the BMI groups. Meller et al. found higher rates of systemic complications (e.g., pneumonia, thromboembolic events, or renal failure) only in morbidly obese (BMI > 40 kg/m^2^) and super-obese (BMI > 50 kg/m^2^) patients [20]. Our overall complication rate was higher than in elective hip surgery but comparable with other studies in a similar German cohort [25]. However, BMI could not be verified as a risk factor for early postoperative complications. Comparable studies by Batsis et al. [12], Tucker et al. [18], and Bryant et al. [24] presented patient collectives with OB patient rates of 8.8%, 21%, and 29%, respectively. Shaparin et al. described an obesity rate of 45%, with 18% reaching obesity grades II or III (BMI > 35 kg/m^2^). All of these studies had their origin in the United States of America. In our German study population, the obesity rate was 6.2%, with only 1.6% reaching BMI values > 35 kg/m^2^. This corresponds with the latest publications by the U.S. Center for Disease Control and Prevention, which estimated an obesity rate among adults of 42.4% [26]. In Germany, only 18.1% of adults suffer from obesity [27]. BMI values above 40 kg/m^2^ are defined as morbid obesity. According to Chee et al., the rate of complications is up to four times higher (22% vs. 5%) compared to non-obese patients, especially for these extreme BMI values [28]. In our patient collective, there were only five (0.53%) morbidly obese patients. The role of obesity in regard to mortality after trauma is controversially discussed in recent literature. 

Differences were seen in early functional outcomes measured by the Barthel index and the ability to walk on crutches. Both parameters showed the best results in the NW and OW patients. Other studies investigated the early and long-term mobilization and functionality after hip arthroplasty. Our results are in line with Bryant et al., showing a faster “out of bed” mobilization for NW and OW patients [24], while Busato et al. reported better mobility in NW and OW patients [29]. Both studies showed a linear decrease of functionality with minimally higher BMI levels. Our results suggested a reversed J-curve pattern with regard to mobility rather than a linear decrease, as described previously [30]. It appears that in the early postoperative period, patients with moderately elevated BMI (25–30 kg/m^2^) presented better functional outcomes. However, BMI > 30 kg/m^2^ was associated with decreased functionality. Nevertheless, this reversed J-curve relation seems to apply only to the early postoperative phase. Modig et al. assessed the relationship between BMI and the chance to return to living at home four months postoperatively. They found that underweight people had a significantly lower likelihood of returning to their home and could not prove a disadvantage for obese patients [31]. The initial disadvantage of delayed mobilization in obese patients is not seen until four months post-operation.

The patients in our study had a mean age of 74.8 years and were predominantly (67%) female. These findings are in concordance with the data for comparable studies [32]. The mean BMI of 23.5 kg/m^2^ in our population was less than the expected value when compared to a German Register Study from 2017. The age-adapted mean BMI of elderly people between 70 and 75 years old in Germany was 26.3 kg/m^2^. In our study, the distribution of the BMI groups showed an overrepresentation of UW patients (8.4% vs. 1.8%) and fewer OB patients when compared to the German national average (6.2% vs. 18.4%) [33], being that UW is a known risk factor for sustaining a proximal femur fracture. Therefore, it is not surprising that we found more UW patients in our study [8,34]. The higher risk of falling and decreased bone mineral density (BMD) in sarcopenic patients appear to be the causes [6,7]. The higher rate of osteoporosis in UW patients as compared to OB patients (12.5% vs. 8.5%) can be reproduced from our data (Table 3). One explanation might be the protective effect of increased trochanteric soft tissue shielding the bone in OB female patients with hip fracture [35].

Our study supports other results identifying more femoral neck fractures (53%) than trochanteric fractures (47%) [4]. Our data also confirms previous findings that those with increasing age are more likely to suffer a trochanteric fracture than a femoral neck fracture [3,36]. Interestingly, we found more trochanteric fractures among OB patients. The distribution of the fracture type depending on the BMI group was J-curved. UW patients had a 50% risk of sustaining a trochanteric fracture. NW patients had the lowest risk (44.7%) and OB patients (64.4%) had the highest risk of sustaining a trochanteric fracture. This is in contrast with former studies where trochanteric fractures were more associated with lower body weight [36]. Logistic regression confirmed these findings of high BMI as an independent risk factor adjusted for age and gender. The lower prevalence of osteoporosis in OB patients [37] was expected to decrease the risk of trochanteric fractures. Against former assumptions of a linear and positive effect of BMI on BMD, newer data show that BMD decreases in obese and morbidly obese patients [38]. Mice fed a high-fat diet developed lower bone density and higher trabecular separation compared to mice on a normal diet [39]. Adipose tissue itself seems to have a negative influence on bone quality [40]. Irving et al. described a shift of the fracture pattern towards more comminuted fractures, especially for OB patients, in trochanteric fractures. A biomechanical influence of the cushioning of trochanteric soft tissue is considerable [41].

Femoral neck fractures showed significantly (*p* = 0.045) higher in-hospital mortality (4.3%) compared to trochanteric fractures (2.0%). This is surprising, especially as patients with trochanteric fractures are older compared to patients with femoral neck fractures (76.1 ± 13.3 years vs. 73.7 ± 13.6 years). The higher mortality of femoral neck fractures might be due to longer operation times, higher rates of blood transfusion, and additional risk during cement application. However, our findings are in contrast with the results from Fox et al. who found higher mortality (including in-hospital mortality) for trochanteric fractures after 2, 6, and 12 months [3].

Revision surgery was necessary in 8.4% of all patients, with OB patients showing an increased but not statistically significant tendency to require the most revisions. Another investigation involving a large register study showed an increased revision rate for OB patients [42]. Sayed-Noor et al. found higher 2-year and 5-year revision rates for OW and OB patients in primary THA. The most common indication for revision was infection [43]. Our revision rate was higher than the comparable literature. Dy et al. found an overall revision rate of 5% after THA, particularly if they were fracture-related arthroplasties [44]. 

Several studies have shown an increased risk for wound site infections for OB patients [45,46]. Impaired perfusion because of decreased capillary density and altered cellular immune competence due to a chronic low-grade inflammatory process were some of the discussed reasons [47]. However, we could not reproduce these findings. The rates of surgical site infections were higher in the OW and OB patients but did not reach statistical significance. Of the 425 patients treated with arthroplasty, 2.4% suffered secondary joint dislocation. Several authors have stated a higher risk of arthroplasty dislocation for OB patients [48]. We found marginally more dislocations in OB patients (3.4%) compared to the mean of our cohort (2.4%). This effect is thought to be caused by a lateralizing force resulting from an impingement phenomenon because of thigh-to-thigh contact in OB patients [49]. Some workgroups have detected problems in positioning prostheses in OB patients. Mainly, the positioning of the socket seems to be challenging and may lead to higher rates of luxation [50].

Existing literature investigating blood loss during hip arthroplasty in OB patients shows an increase in necessary transfusion rates with higher body weight [51]. We could not reproduce these results. In contrast, OB patients received less erythrocyte concentrate transfusions than the other three groups. Cao et al. recently published results that confirm our observations [52]. The higher total blood volume in OB patients seems to result in a lower volume of relative blood loss [53]. As an interfering factor, there is malnutrition, which leads to low BMI and undetected anemia, resulting in a higher risk of perioperative transfusion [54]. Preoperative anemia was not assessed in our data.

In our investigations, there were no significant differences between the BMI groups regarding mortality. Surprisingly, the mortality rate for OB patients was lower than in any other BMI group. This fact is in accordance with findings from Alban et al. and Evans et al. [55,56]. Modig et al. also demonstrated this effect. In a large register study they confirmed the obesity paradox of obese patients having significantly higher 1-year survival rates after proximal femur fractures [31].

## 5. Conclusions

In conclusion, there are differences in some outcome parameters in patients sustaining proximal femoral fractures regarding their preoperative BMI. Longer operation times and longer LOS are the key findings. Trends in our data also show that OB patients may be at higher risk for postoperative complications, such as wound infections, need for revision, or even systemic complications (e.g., urinary tract infections and pneumonia). This association is even more distinct in severely obese patients with BMI values > 35 or 40 kg/m^2^.

## 6. Limitations

The principal limitations of our study are due to its retrospective study design and the long period of data collection. All of the information about complications, as well as body weight and height, came from the medical files of patients. The information was not captured by a conscientious investigator in a prospective study. Furthermore, there were several different surgical techniques and surgeons that may have affected the results. As a single-center study, our results can only be applied to the population in Munich, Bavaria. In other regions with a higher proportion of OB patients, the differences between BMI groups might be even more distinctive.

## Figures and Tables

**Table 1:** Baseline characteristics and outcomes depending on fracture type.

	Femoral Neck Fracture	Trochanteric Femur Fracture	*p*-Value
*n* (%)	506 (53%)	444 (47%)	
Age (years)	73.7 ± 13.6	76.1 ± 13.3	0.002 *
Gender female	345 (68.2%)	292 (65.8%)	0.429
BMI (kg/m^2^)	23.3 ± 3.8	23.8 ± 4.6	0.274
ASA score	2.3 ± 0.7	2.3 ± 0.7	0.239
Osteoporosis	39 (7.7%)	40 (9.0%)	0.469
Length of stay (days)	13.8 ± 10.0	13.8 ± 7.5	0.294
Mortality	22 (4.3%)	9 (2.0%)	0.045 *
Mobility on crutches	298 (63.0%)	212 (51.2%)	<0.001 *

Data presented as mean ± SD or *n* (%); * = statistically significant; BMI = body mass index.

**Table 2:** Logistic regression analysis of independent risk factors for trochanteric fracture rather than femoral neck fracture adjusted for the covariates age, gender, and osteoporosis.

	OR	95% CI	*p*-Value
BMI	1.033	1.002–1.066	0.040 *
Age	1.016	1.006–1.027	0.002 *
Gender female	1.243	0.932–1.658	0.138
Osteoporosis (anamn.)	1.202	0.752–1.922	0.441

Note: * = statistically significant; OR = odds ratio; CI = confidence interval; BMI = body mass index; anamn. = anamnestically reported.

**Table 3:** Epidemiology and history of comorbidities.

	Under-Weight (*n* = 80)	Normal Weight (*n* = 570)	Over-Weight (*n* = 241)	Obese (*n* = 59)	*p*-Value
Age (years)	76.9 ± 15.1	74.8 ± 13.9	74.5 ± 12.1	72.8 ± 12.8	0.089
Gender female	66 (82.5%)	392 (68.8%)	140 (58.1%)	39 (66.1%)	<0.001 *
ASA score	2.27 ± 0.7	2.25 ± 0.7	2.31 ± 0.7	2.54 ± 0.7	0.012 *
Fracture type					0.034 *
Femoral neck fracture	40 (50%)	315 (55.3%)	130 (53.9%)	21 (35.6%)	
Trochanteric fracture	40 (50%)	255 (44.7%)	111 (46.1%)	38 (64.4%)	
N^o^ of preexisting morbidities	2.47 ± 1.9	2.38 ± 1.8	2.58 ± 1.8	3.10 ± 1.6	0.004 *
Diabetes mellitus	7 (8.8%)	78 (13.7%)	57 (23.7%)	22 (37.3%)	<0.001 *
Arterial hypertension	32 (40.0%)	288 (50.5%)	145 (60.2%)	44 (74.6%)	<0.001 *
Coronary heart disease	20 (25.0%)	148 (26.0%)	76 (31.5%)	24 (40.7%)	0.053
Hypothyroidism	11 (13.7%)	75 (13.2%)	31 (12.9%)	13 (22.0%)	0.302
Osteoporosis	10 (12.5%)	49 (8.6%)	15 (6.2%)	5 (8.5%)	0.331
Dementia	15 (18.8%)	70 (12.3%)	24 (10.0%)	5 (8.5%)	0.186
Stroke	7 (8.8%)	58 (10.2%)	28 (11.6%)	6 (10.2%)	0.898
Substance addiction	13 (16.3%)	37 (6.5%)	13 (5.4%)	2 (3.4%)	0.004 *
Chronic kidney disease	6 (7.5%)	48 (8.4%)	28 (11.6%)	9 (15.3%)	0.201
Tumor disease	16 (20.0%)	78 (13.7%)	32 (13.3%)	9 (15.3%)	0.466
N^o^ of medications	3.4 ± 3.5	3.5 ± 3.4	3.9 ± 3.5	4.4 ± 3.0	0.103

Underweight: BMI < 18.5 kg/m^2^; normal weight: BMI 18.5–24.9 kg/m^2^; overweight: BMI 25.0–29.9 kg/m^2^; obese: BMI ≥ 30.0 kg/m^2^. Data presented as mean ± SD or *n* (%); * = statistically significant; ASA = American Society of Anesthesiologists.

**Table 4:** Complications and outcome variables.

	Under-Weight (*n* = 80)	Normal Weight (*n* = 570)	Over-Weight (*n* = 241)	Obese (*n* = 59)	Bivariate *p*-Value ^a^	Multivariate *p*-Value ^b^
Revision	4 (5.0%)	47 (8.2%)	22 (9.1%)	7 (11.9%)	0.512	0.538
Erythrocyte transfusion	30 (37.5%)	195 (34.3%)	78 (32.4%)	19 (32.8%)	0.852	0.873
Wound site infection	1 (1.3%)	14 (2.5%)	9 (3.7%)	3 (5.1%)	0.422	0.511
Urinary tract infection	6 (7.5%)	51 (8.9%)	23 (9.5%)	6 (10.2%)	0.940	0.810
Pneumonia	4 (5.0%)	24 (4.2%)	12 (5.0%)	4 (6.8%)	0.817	0.751-
Deep vein thrombosis	1 (1.3%)	7 (1.2%)	4 (1.7%)	0 (0.0%)	0.953	0.980-
Operation time (min)	85.3 ± 42.9	90.2 ± 38.2	99.9 ± 39.9	117.2 ± 61.5	<0.001 *	<0.001 *
Mobility on crutches	34 (45.9%)	310 (58.6%)	138 (59.5%)	28 (53.8%)	0.098	0.481
Barthel Index score	41.7 ± 19.5	46.6 ± 22.3	48.9 ± 17.1	45.0 ± 12.2	0.062	0.052
Mortality	2 (2.5%)	17 (3.0%)	11 (4.6%)	1 (1.7%)	0.065	0.628
Length of stay (LOS)	12.3 ± 4.8	13.6 ± 7.8	14.2 ± 11.7	16.0 ± 9.7	0.040 *	0.040 *

Underweight: BMI < 18.5 kg/m2; normal weight: BMI 18.5–24.9 kg/m2; overweight: BMI 25.0–29.9 kg/m2; obese: BMI ≥ 30.0 kg/m^2^. Data presented as mean ± SD or n (%); * = statistically significant; ^a^ bivariate analysis: Chi-square test for binary and Kruskal–Wallis test for continuous variables; ^b^ multivariate analysis adjusted for age, gender, ASA score, and fracture type; logistic regression for binary variables and linear regression for continuous variables.

**Table 5:** Linear regression for length of stay (log.) and operation time (log.) over BMI categories adjusted for age, gender, ASA score, and fracture type.

	Length of Stay (LOS)			Operation Time		
	OR	95% CI	*p*-Value	OR	95% CI	*p*-Value
BMI underweight	−0.210	−0.358–−0.066	0.004 *	−0.320	−0.457–−0.183	<0.001 *
BMI normal	−0.128	−0.244–0.013	0.029 *	−0.253	−0.363–−0.144	<0.001 *
BMI overweight	−0.113	−0.235–0.010	0.071	−0.144	−0.260–−0.028	<0.001 *
BMI obese	*reference*			*reference*		
Age	0.006	0.003–0.008	0.002 *	−0.003	−0.005–−0.001	0.008 *
Gender female	−0.043	−0.018–0.103	0.164	−0.017	−0.075–0.040	0.552
ASA score	0.116	0.076–0.155	<0.001 *	−0.002	−0.040–0.035	**0.899**
Fracture type	0.002	−0.052–0.057	**0.931**	−0.253	−0.305–−0.201	<0.001 *

Note: * = statistically significant; log. = logarithmized variable; OR = odds ratio; CI = confidence interval; BMI = body mass index.

## Abbreviations

ASA	American Society of Anesthesiologists
BMI	Body mass index
DHS	Dynamic hip screw
DVT	Deep vein thrombosis
FNF	Femoral neck fracture
HA	Hemiarthroplasty
ICD-10	International Statistical Classification of Diseases and Related Health Problems (10th revision)
LOS	Length of stay
NW	Normal weight
OB	Obese
OR	Odds ratio
OW	Overweight
TF	Trochanteric fracture
THA	Total hip arthroplasty
TUM	Technical University Munich
UW	Underweight
WHO	World Health Organization
